# Case report: Osteitis/osteomyelitis pubis simulating acute appendicitis

**DOI:** 10.1016/j.ijscr.2018.10.077

**Published:** 2018-11-02

**Authors:** James G. Glasser

**Affiliations:** Janet Weis Children’s Hospital, Geisinger Medical Center, 100N. Academy Ave., Danville, PA 17822, United States

**Keywords:** Athletic (sports) hernia, Osteitis pubis, Osteomyelitis pubis, Acute appendicitis’ differential diagnosis

## Abstract

There is a continuum between Athletic (Sports) Hernia, Osteitis Pubis, and Osteomyelitis Pubis. The pubis is the site of attachment of many “core” muscles. A lay term used to describe a hernia is “rupture”. Athletic hernia denotes a tear. Chronic musculotendinous strain may cause inflammation (osteitis pubis). An inflammatory focus may become a nidus for infection (osteomyelitis pubis). The symptoms caused by these three entities blur one with the other and with those characterizing acute appendicitis. This is an important association for clinicians to know.

## 1 Case report

### 1.1 Presented in accordance with the SCARE guidelines [1]

A 16 years old young man came to Geisinger Emergency Department because of right lower quadrant abdominal pain that was acute in onset and had worsened over the prior two days; it was accompanied by fever, anorexia, nausea, and vomiting.

PMH: He was an accomplished athlete; and his father’s over-riding concern was that his son be evaluated thoroughly and treated expeditiously, so as to resume football practice *as soon as possible*!

**Fig. 1:**
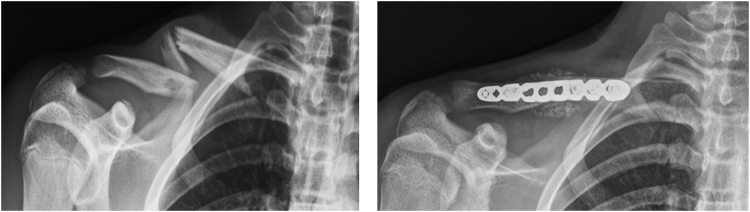
Clavicle fracture 2 years prior to this illness.

Two years ago he had fractured his right clavicle; and because of the poor alignment and delayed healing, operative reduction and fixation was performed (Figure 1).

He was remarkably tender in the right lower quadrant of his abdomen, with involuntary guarding and positive psoas and obturator signs. His WBC count and Urinalysis were normal. Ultrasound identified “mildly prominent, non-specific lymph nodes” but not the appendix. The MRI was also “somewhat equivocal” -free fluid in the pelvis with inflammation, but the appendix was not visualized; nevertheless, the radiologist opined that the findings were consistent with either appendicitis or inflammatory bowel disease. Unfortunately, the MRI did not include the pubic symphysis and contiguous musculature.

The diagnostic impression was appendicitis; however, this was not corroborated by laparoscopy, which revealed a normal appendix. No other intra-abdominal pathology was identified. The presumptive diagnosis became gastroenteritis, and an uneventful recovery was anticipated. This prediction initially appeared correct, and he was discharged only to return the evening of the second post-operative day, complaining once again of exquisite right lower quadrant abdominal pain, this time associated with fever, leukocytosis, and elevated inflammatory markers (Table 1).

**Table 1:** Summary of Lab Data.

Date	WBC	CRP	ESR
Pre-op	4.34		
DOS	4.98	224	28
PO #1	8.84	202	
PO #2	9.0	155	41

**Fig. 2:**
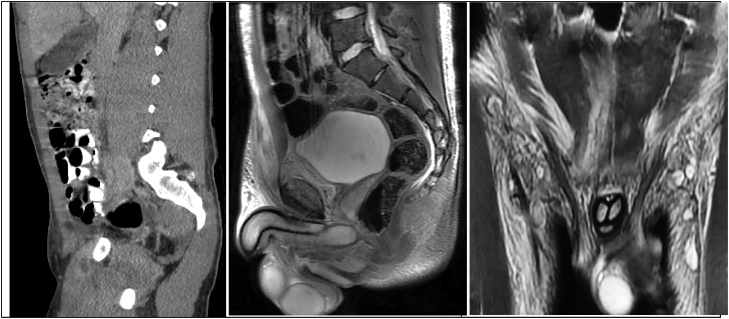
Sagital images show rim enhancing collections anterior and superior to the pubis, the retro-pubic fluid, and blurring of the pubic cortex. The coronal view shows inflammation of the right rectus muscle.

**Fig. 3:**
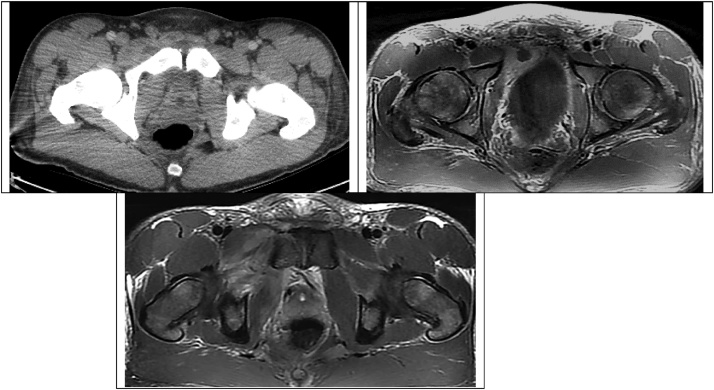
Axial views demonstrate these fluid collections, as well as inflammation of the pectineus muscle.

The second admission’s CT and MRI demonstrate (Fig. 2, Fig. 3):

• Fluid in the retro-pubic space of Retzius
• Two rim enhancing collections within the pectineus and rectus abdominal muscle denoting either myositis or a periosteal abscess
• Blurring of the cortex (growth plate) of the right superior pubic ramus

**Fig. 4:**
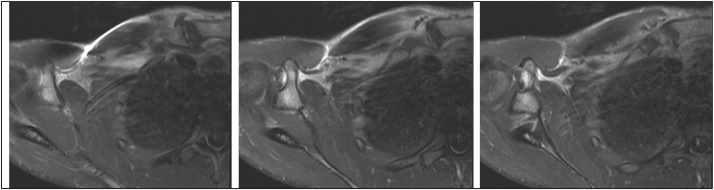
Septic arthritis adjacent to the clavicular fracture site.

Blood cultures were obtained, and he was treated empirically with NSAID’s and antibiotics to cover presumed osteitis pubis or osteomyelitis. Clindamycin was switched to Cefazolin, because the blood culture grew MSSA, resistant to Clindamycin. Clinical improvement was followed by discomfort in his right shoulder, and radiographs disclosed septic arthritis in the sternoclavicular joint (Fig. 4).

The consultant orthopedist explored the sternoclavicular joint and drained approximately 10 ml of purulent material. The joint was irrigated; infected bone and cartilage were curetted; and adjacent soft tissues were debrided. The hardware was not removed, but ultimately this might be necessary.

## 2 Discussion

A**thletic hernia, osteitis pubis,** and **pubic osteomyelitis** constitute a spectrum of disability, resulting from injury and inflammation that may be complicated by infection [2,3]. These patients complain of lower abdominal or groin discomfort that is exacerbated by movement. Usually they have participated in activities that have stressed the lower abdominal, groin, and crural muscles, which attach to the pubis. The pubic symphysis is a non-synovial, amphiarthrodial joint, comprised of fibrocartilage nestled between hyaline cartilage [4].

The anterior “core” muscles attach to the **superior** pubic ramus.

• The pectineus and rectus muscles attach medially.
• The conjoined tendon attaches laterally.

The adductors (longus, brevis, magnus, and gracilis) attach to the **inferior** pubic ramus. These muscle groups are antagonistic and act to stabilize the torso. Repetitive strain may tear these muscles or their tendinous insertions, causing concomitant ileoinguinal nerve injury or osteitis pubis. Infection may supervene and result in osteomyelitis pubis [5,6].

Pubic or groin discomfort occurs in all these entities. The pain is insidious in onset and unremitting. Typically, it is associated with twisting, kicking, or sprinting with sudden changes in direction; these movements characterize numerous sports: basketball and football; soccer, lacrosse, and hockey; even golf, tennis, or kayaking! Treatment entails rest, anti-inflammatory agents (NSAID’s and Steroids), and physiotherapy.

Discomfort that is refractory to conservative modalities suggests a more serious problem:

• A tear of the inguinal canal (external oblique aponeurosis or conjoined tendon) necessitates repair.
• Concomitant ileoinguinal nerve injury may require ablation of the nerve.
• Osteomyelitis requires antibiotics and possibly surgical debridement.

Hematogenous seeding is the most common etiology of osteomyelitis in children. The offending organism is Staph aureus (90% of infections, 50% of which are MRSA). The metaphysis in children has a transphyseal blood supply. Blood vessels pass from the metaphysis, through the epiphysis, into the joint. Bacteria are filtered in the capillaries and adhere to the extracellular bone matrix and cartilage, which are privileged areas where pathogens can evade host defenses and colonizing the bone and attacking the host’s cells [7].

Bacterial invasion may also occur from contiguous soft tissue infection:

• Skin injuries/scratches with cellulitis
• Pustular dermatologic diseases (psoriasis vulgaris, palmoplantar pustulosis, and eczema)

Symptoms of osteomyelitis are acute unremitting groin pain, associated with fever and irritability; and occasionally, inflammation of the overlying soft tissues. There may be suprapubic tenderness, and pain upon hip abduction that radiates towards the groin or testes. Usually, the WBC count is increased with PMN’s predominating. Inflammatory markers (ESR and CRP) are elevated, also.

Trauma (with contamination) occurs in 30% of patients with osteomyelitis:

• Surgical manipulations, such as laparoscopic herniorrhaphy, in which a prosthesis is attached to the pubis;
• And penetrating wounds.

**Treatment** is a six weeks’ course of antibiotics. Clinical deterioration, while on antibiotics, mandates surgical intervention, as in the case presented.

## 3 Conclusion

This case illustrates two things:

• There is a continuum among diagnostic entities previously considered disparate: athletic hernia and osteitis pubis, and osteomyelitis pubis.
• These diagnostic entities may simulate an acute abdomen.

It is a truism that “we see what we look for”. In the same way that right lower lobe pneumonia simulates an acute abdomen, and irritation of the diaphragm simulates shoulder injury, the above entities may simulate acute appendicitis [8].

## Conflicts of interest

None.

## Sources of funding

None.

## Ethical approval

IRB approval is not necessary in case reports consisting of only one patient.

No personal identifying information is disclosed in this case report.

The patient’s mother consented to the preparation of this case report.

## Consent

Only when more than three patients are involved in a report is IRB approval required by

Janet Weis Children’s Hospital, Geisinger Medical Center.

The patient’s mother consented to the submission of this case report.

## Author contribution

I am the only person who contributed to the preparation of this report.

## Registration of research studies

NA.

## Guarantor

James G. Glasser, MD, FACS, Old Shell Road, Mobile, AL 36607, jasgler@gmail.com.

## Authorship

The author attests that he meets the current ICMJE criteria for authorship.

## Provenance and peer review

Not commissioned, externally peer reviewed.
